# Defining the viability of tardigrades with a molecular sensor related to death

**DOI:** 10.1371/journal.pone.0206444

**Published:** 2018-10-26

**Authors:** Myriam Richaud, Simon Galas

**Affiliations:** IBMM, University of Montpellier, CNRS, ENSCM, Montpellier, France; University of Colorado Boulder, UNITED STATES

## Abstract

The design of experimental protocols that use animal models to assess the impact of a stress on a population or to determine the life span expectancy impact can be time-consuming due to the need for direct observations of dead and living animals. These experiments are usually based on the detectable activity of animals such as food intake or mobility and can sometimes produce either under- or overestimated results. The tardigrade *Hypsibius exemplaris* is an emerging model for the evolutionary biology of the tardigrade phylum because of its convenient laboratory breeding and the recent introduction of new molecular tools. In this report, we describe the use of a new fluorescent dye that can specifically stain dead tardigrades. Furthermore, we also monitored the absence of a toxic side effect of the death-linked fluorescent dye on tardigrade populations. Finally, we conclude that tardigrade experiments that require survival counting of the *Hypsibius exemplaris* species can be greatly improved by using this technique in order to limit underestimation of alive animals.

## Introduction

With a length of 0.1–1.2 mm, tardigrades can inhabit seas, fresh water or the water films of terrestrial moss and lichens [1]. However, the main reason for the interest in tardigrades lies in their capacity to cope with the harshest treatments or to withstand deleterious environmental conditions. For example, they can survive a 10 kJ/m^2^ UV exposure [2], a ten-day space flight exposure to solar radiation at low earth orbit in the space vacuum [3], and an exposure of up to 7.5 GPa [4], a pressure equal to that prevailing at a depth of up to 180 km from the earth surface. Moreover, tardigrades can resist harsh treatments with organic solvents [5], extreme temperatures (ranging from -272 to 151°C) or high radiation dose (kGy) intensities [6]. The tardigrade can enter an anhydrobiotic latent life state called “tun” that forms by dehydration. Some marine or terrestrial tardigrade species share the capacity to turn on anhydrobiosis. However the anhydrobiosis can be principally encountered in the terrestrial tardigrade group. This is mainly under this inactive and ametabolic anhydrobiotic state [7] that tardigrade can cope with the harshest environmental conditions [8–11].

Up to 1000 species of tardigrade have been reported [12,13,14], but few species can be routinely maintained in the laboratory for experimental investigations. The *Hypsibius exemplaris* (previously known as *H*. *dujardini*) species is a limnetic tardigrade species that can enter anhydrobiosis when its surrounding water film progressively vanishes. *H*. *exemplaris* is considered an emerging model for studies on the evolutionary developmental biology of tardigrades. The genome of *H*. *exemplaris* has recently been sequenced [15–19], and the laboratory culture protocols [20] as well as new genetic knockdown techniques by RNA interference (RNAi) have been described [21]. The *H*. *exemplaris* species does not show the higher tardigrade resistance to the harshest stress treatments. However, because of the growing number of techniques now available for *H*. *exemplaris*, we found it interesting to develop new tools to facilitate the viability assay protocols with this emerging laboratory model species of tardigrade.

It is known that *H*. *exemplaris* specimens can be marked as living when a corresponding motility or feeding behavior can be reported by a direct observation of the culture. However, this species sometimes fails to show any motility, for example, when a molt occurs or if individuals prepare egg laying. In such cases, the scoring of dead animals can be more time-consuming than necessary. To avoid such experimental problems and to improve the tools offered by this emerging model, we assessed whether an indirect observation that specifically marks dead animals can be developed.

We then assessed whether the SYTOX Green Nucleic Acid stain, a new fluorescent dye, may highlight dead animals in cultures of the *H*. *exemplaris* tardigrade. We show that SYTOX green does not have detectable toxicity and can mark dead tardigrades from 1 h to 6 days after labelling, without affecting the normal tardigrade survival. Moreover, we also observed that the living tardigrade scoring by the SYTOX green technique can limit survival underestimation when compared to a classical examination by direct counting.

We conclude that experimental assays such as lifespan measurement, stress treatments, effect assessments, or rehydration of anhydrobiotic animals can be greatly improved by the use of SYTOX green labelling with the *H*. *exemplaris* tardigrade.

## Results

### Sodium azide impacts the viability of *Hypsibius exemplaris*

**Fig 1:**
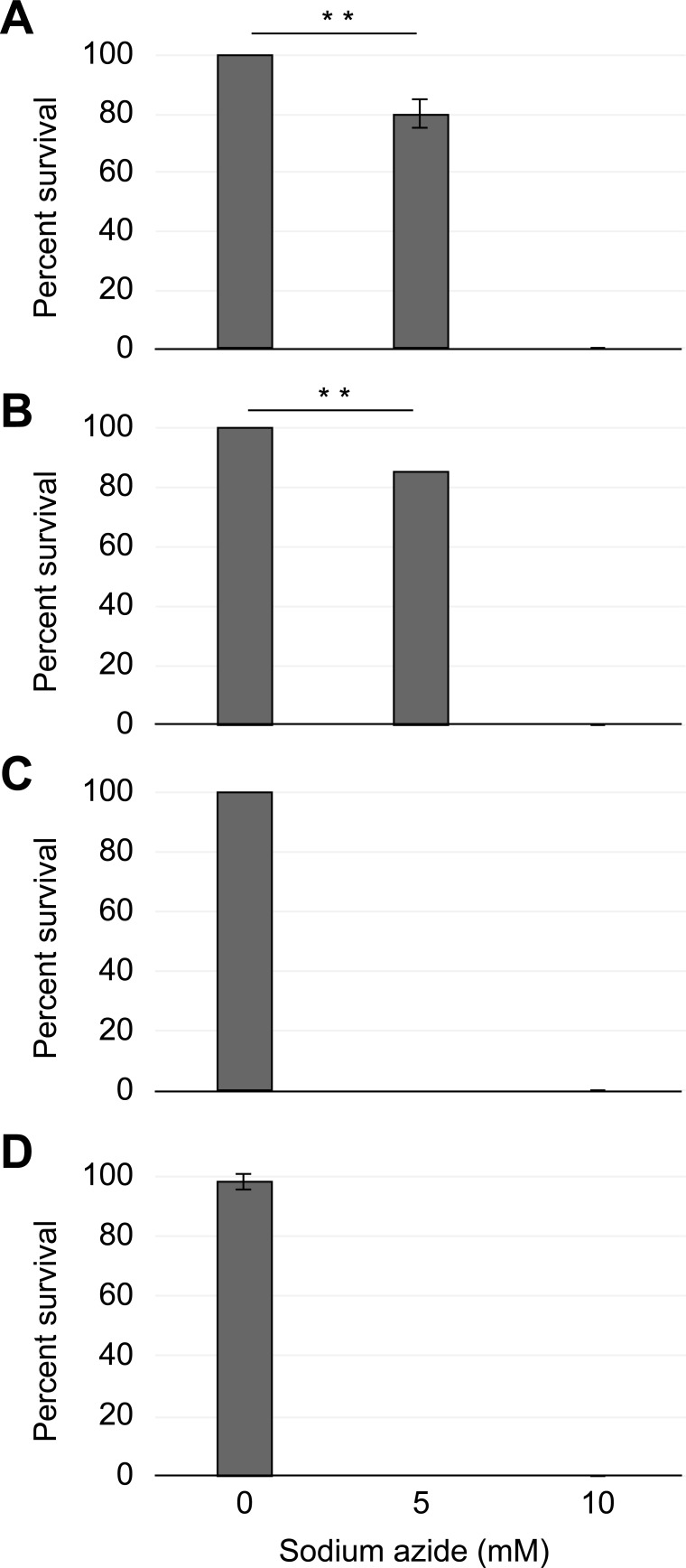
Direct scoring of the tardigrade viability reveals the highly toxic effect of sodium azide. The average fraction of living tardigrades (y-axis) treated with either 0, 5 or 10 mM sodium azide concentration (x-axis) and scored by direct observation at 1 h (A), 24 h (B), 48 h (C) or 6 days (D) after the experiment began. Error bars indicate the standard deviation from at least three experiments, and a double asterisk represents the respective significant difference at α = 0.01 (Z-test) degree. A complete description of statistical results is indicated in the text.

Groups of active tardigrades were incubated with either 5 or 10 mM sodium azide, and their viability was monitored by direct counting of living animals. As shown in Fig 1, a treatment with 5 mM sodium azide induced a highly significant fraction (Z-test, α = 0.01, *p*-value = 0.000) of dead animals in comparison with the controls, when the observation was made immediately after drug addition (Fig 1A), while the treatment of tardigrade groups with 10 mM sodium azide resulted in their immediate death. Furthermore, the fraction of dead tardigrades remained roughly the same when the observation of the 5 mM sodium azide-treated group was made at 24 h (Fig 1B), and the corresponding statistical analysis revealed a highly significant difference in comparison with the control group (Z-test, α = 0.01, *p*-value = 0.001). The direct counting of living tardigrades performed at either 48 h or 6 days did not reveal any living tardigrades in both the 5 and 10 mM sodium azide-treated groups (Fig 1C and 1D).

It is interesting to observe that up to 80% of the *H*. *exemplaris* tardigrade groups submitted to 5 mM sodium azide could cope with the treatment for up to 24 h after the beginning of the treatment (Fig 1B).

### The SYTOX green dye does not impair tardigrade viability

In the previous section, we used a sodium azide treatment that allowed us to trigger reproducible tardigrade deaths in a controlled manner. With the goal of substituting the direct scoring technique of tardigrade survival by a death-linked and specific dye, we then assessed whether a treatment with the fluorescent SYTOX green dye may not impair, by itself, the tardigrade groups’ viability.

**Fig 2:**
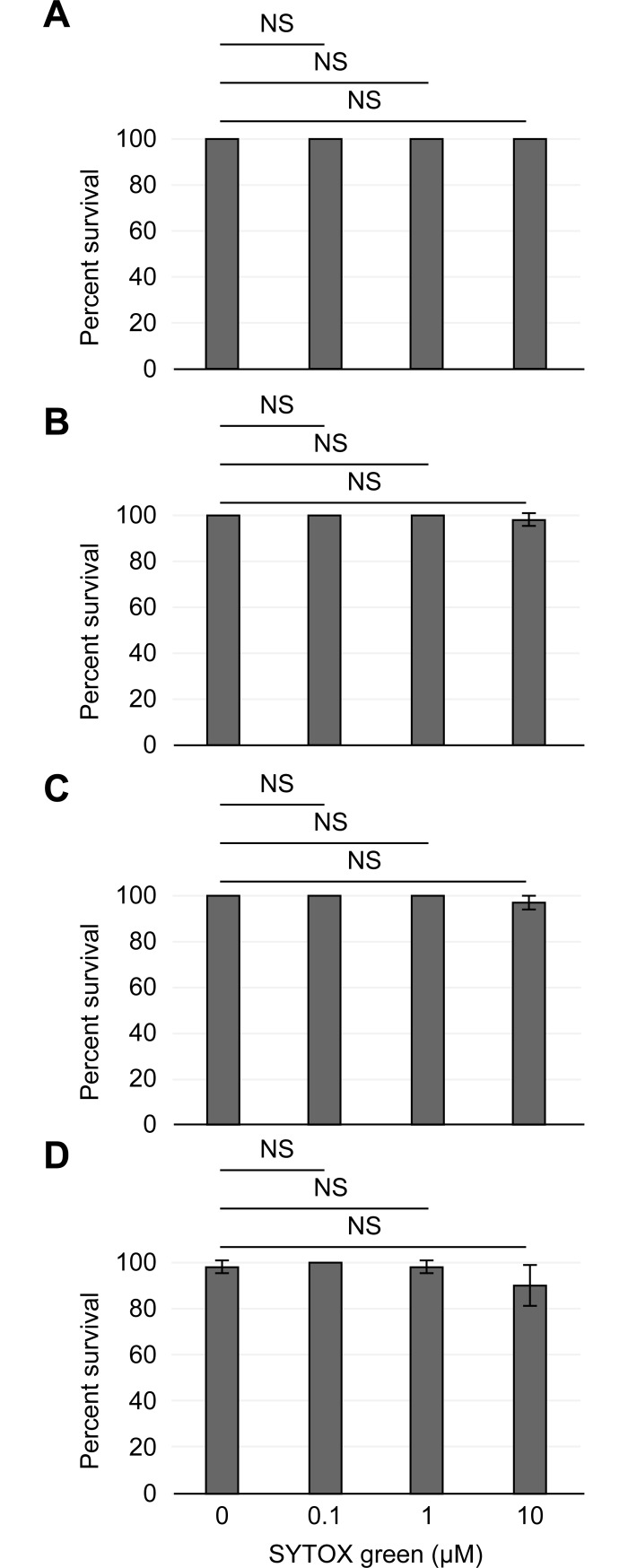
The SYTOX green dye is not toxic for tardigrades. The average fraction of living tardigrades (y-axis) treated with increasing SYTOX green dye concentrations (0, 0.1, 1 or 10 μM; x-axis) and scored by direct observation of viability at either 1 h (A), 24 h (B), 48 h (C) or 6 days (D) after the beginning of the experiment. Error bars indicate the standard deviation from at least three experiments and the “NS” indicates a non-significant difference at α = 0.05 degree (Z-test). A complete description of statistical results is indicated in the text.

To do so, tardigrade groups were incubated with either 0.1, 1 or 10 μM SYTOX green, and living animals were scored by direct counting. As shown in Fig 2, tardigrade survival was not affected by treatment with the SYTOX green dye at either 1 h (Fig 2A) or 24 h (Fig 2B) of incubation. We then assessed whether the SYTOX green dye could possibly exert a delayed toxic effect on the tardigrade groups. Fig 2C shows the viability of the tardigrade groups at 48 h after the SYTOX green dye addition to the culture media. We were not able to detect a decrease in the tardigrade viability. Furthermore, the tardigrade viability was not significantly impaired (Z-test, α = 0.05, *p*-value = 1.000) for all of the experimental groups in up to 6 days of incubation with the three SYTOX green concentrations assessed (Fig 2D).

Thus, in light of the results described in this section, we can conclude that the SYTOX green does not impair the viability of the *H*. *exemplaris* tardigrade. In addition, we also demonstrated the possibility of adding the SYTOX green dye directly at the beginning of a viability assay without any subsequent alteration of the tardigrade survival.

We next wondered about the most suitable SYTOX green dye concentration that may be associated with the optimal scoring of dead tardigrades with the least interference. Because we did not detect any interference with tardigrade survival by the SYTOX green dye at all of the concentrations that we assessed (Fig 2A–2D), we then decided to confirm the use of the fluorescent dye at the three concentrations that we assessed by a death-linked fluorescence scoring.

### The SYTOX green dye improves the tardigrade viability determination

As we showed in the previous section, the SYTOX green incubation did not have a negative impact on the tardigrade viability. We then assessed whether the SYTOX green dye association with the sodium azide treatment may allow the detection of the survival fraction of tardigrades by means of the death-linked fluorescent signal.

**Fig 3:**
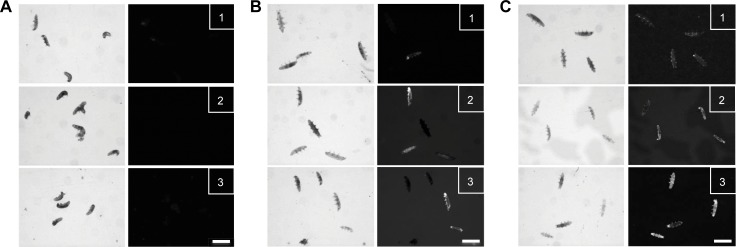
The SYTOX green fluorescent dye can specify the dead tardigrades. Representative photographs of the tardigrade groups 1 h after exposure beginning with 0 mM (A), 1 mM (B) or 10 mM (C) sodium azide and revealed by the linked fluorescence of the SYTOX green dye at a final concentration of 0.1 μM (1), 1 μM (2) or 10 μM (3) SYTOX green dye. The left column shows representative light microscope pictures while right column corresponds to representative fluorescent pictures that highlight the Sytox Green dye linked fluorescence. Scale bar represents 0.1 mm.

Fig 3A shows representative pictures of tardigrade groups after 1 h of incubation with either 0.1 (Fig 3A-1), 1 (Fig 3A-2) or 10 μM (Fig 3A-3) SYTOX green. We could observe the tardigrades under brightfield illumination, but as expected, we did not detect an associated SYTOX green fluorescence because of the absence of sodium azide in the media. We next assessed if a 5 mM sodium azide concentration added to the tardigrade media may induce detectable death-linked fluorescence by the SYTOX green dye. As shown in Fig 3B, we were able to detect a death-linked fluorescence from the dose of 1 μM (Fig 3B–1) of the fluorescent dye. Moreover, the incubation with either 1 μM (Fig 3B-2) or 10 μM (Fig 3B-3) SYTOX green resulted in an improved result because of increased fluorescent staining of the detectable deaths. This last observation was therefore confirmed with a 10 mM sodium azide treatment (Fig 3C) that resulted in a faint SYTOX green dye staining when the tardigrades were incubated with 0.1 μM (Fig 3C-1) SYTOX green. However, the tardigrade groups treated with 10 μM sodium azide showed an increase in the observable death-linked fluorescence when either 1 μM (Fig 3C-2) or 10 μM (Fig 3C-3) SYTOX green was added to the tardigrade media. A brief comparison of the fluorescent staining between the tardigrade groups shown in Fig 3B-1 and both Fig 3B-2 and 3B-3, as well as between Fig 3C-1 and both Fig 3C-2 and 3C-3, allowed us to conclude that 0.1 μM SYTOX green may underestimate the death fraction in tardigrade groups.

Because we observed a comparable death-linked SYTOX green fluorescent staining with both 1 μM (Fig 3B) and 10 μM (Fig 3C) media concentration, we then decided to perform the next experiment with 1 μM SYTOX green to optimize the fluorescent dye volume needed for the experiments.

**Fig 4:**
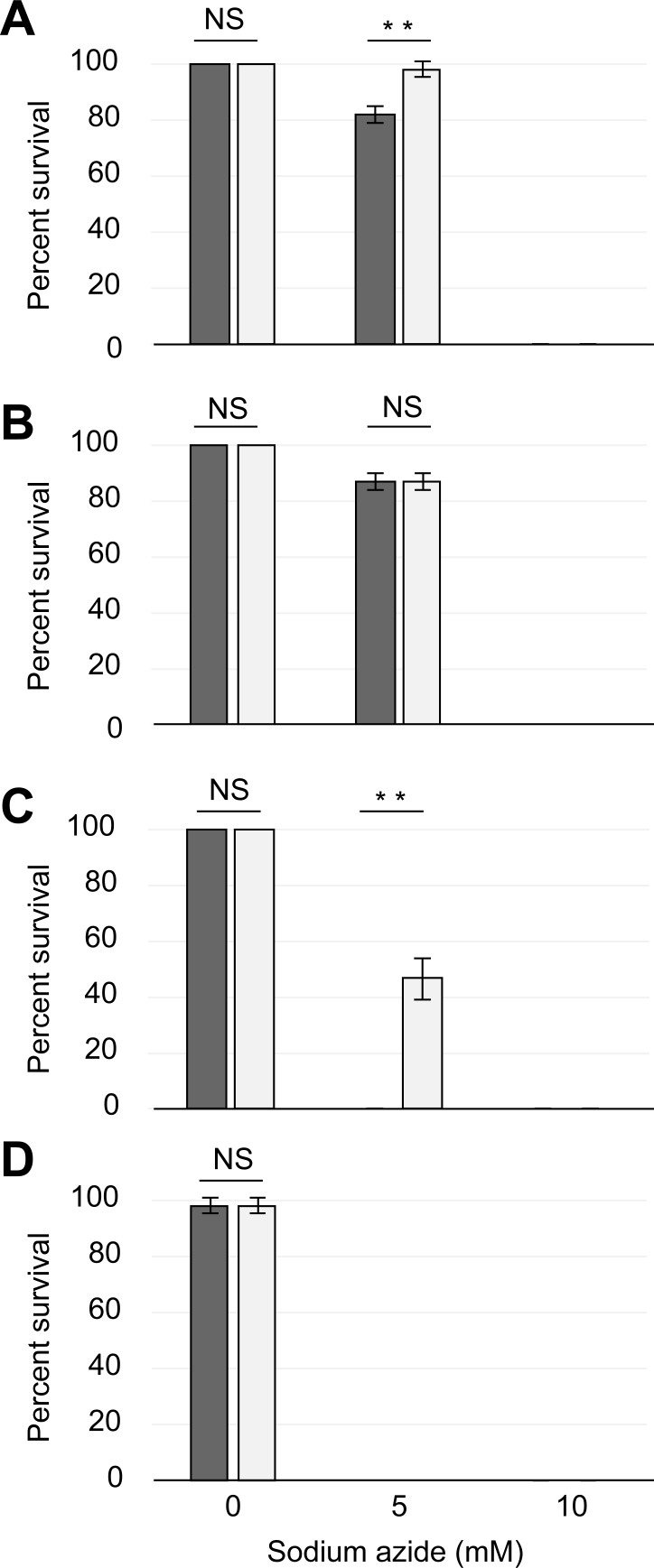
The SYTOX green fluorescent dye can improve the detection of dead tardigrades. The average fraction of living tardigrades treated with either 0, 5 or 10 mM sodium azide and scored by direct observation on brightfield illumination (colored bars, left) or SYTOX green dye linked fluorescence at 1 μM on an epifluorescence microscope (white bars, right), at either 1 h (A), 24 h (B), 48 h (C) or 6 days (D) after the beginning of the experiment. Error bars indicate the standard deviation from at least three experiment repetitions and a double asterisk represents the respective significant difference at α = 0.01 (Z-test) degree, while “NS” indicate a non-significant difference at α = 0.05 degree (Z-test). A complete description of statistical results is indicated in the text.

We next decided to evaluate the relevance of a scored death tardigrade fraction by the SYTOX green fluorescence in comparison with a classical examination by direct counting. Fig 4 represents the viability of the tardigrade groups exposed to either 0, 5 or 10 mM sodium azide and stained with 1 μM SYTOX green fluorescent dye. Fig 4A shows the living tardigrade counts 1 h after the beginning of the experiment. As expected, we observed a non-significant statistical difference (Z-test, α = 0.05, *p*-value = 1.000) between control tardigrade groups scored by either direct observation or the death-linked fluorescence. However, we noted a highly significant statistical difference (Z-test, α = 0.01, *p*-value = 0.000) between both tardigrade groups when 5 mM sodium azide was added to the media. Furthermore, we were not able to detect tardigrade survival by using either one of the two scoring techniques when 10 mM sodium azide concentration was added to the tardigrade media. It is interesting to note that this result matches with the previous observation of Fig 1A in the previous section. This result is of great significance because it challenges the relevance of the classical counting technique by direct observation and uncovers its underestimation bias. More precisely, it is possible that direct scoring of death tardigrade, which is based only on the apparent motility of animals only, must overestimate the total score of death animals when they do not move at all but are always alive.

Fig 4B shows an experiment identical to the previous one but scored at 24 h for tardigrade survival instead of 1 h. We did not observe a significant difference (Z-test, α = 0.05, *p*-value = 1.000) in the tardigrade viability estimation by the two scoring techniques that we used when 0 mM azide was added to the media. Moreover, the 5 mM sodium azide-treated groups also revealed a non-significant difference between both scoring techniques (Z-test, α = 0.05, *p*-value = 1.000). This result may indicate a stabilization of the tardigrade population’s ability to cope with the sodium azide toxicity at this duration of experimental incubation. Interestingly, in the previous section, we noticed a comparable tardigrade viability upon identical sodium azide treatment as shown in Fig 1B.

Fig 4C shows the tardigrade viability at 48 h of incubation. Unexpectedly, we noted a highly significant difference (Z-test, α = 0.01, *p*-value = 0.000) in the observed viability between the tardigrade groups scored by either direct counting or the death-linked fluorescence techniques when 5 mM sodium azide was added to the media. This observation argues for the possibility of a tardigrade death overestimation by the direct counting technique as we previously noticed in Fig 4A because a scoring of the living animals on the basis of their motility can also include living animals that do not move and can be scored as dead.

Fig 4D shows the tardigrade groups’ viability at 6 days of incubation. As expected and in accordance with the results shown in Fig 1D, we were not able to detect any tardigrade survival for either 5 or 10 mM sodium azide incubation of tardigrade groups.

## Discussion

Experimental setups dedicated to tardigrade viability assessments can be time-consuming because of the need for direct scoring of living and dead animals. Such direct scoring of living tardigrades is critical for anhydrobiotic exit survival [22,23] or stress resistance assessments [2,24–26].

In this report, we assessed if an indirect measurement of tardigrade viability may help to discriminate between living and dead tardigrades. To do so, we used SYTOX green, a fluorescent dye that is believed to mark only dead cells [27,28] or death processes of few organisms [29–33] and is used for high content screening of molecules acting on animal survival [34]. The SYTOX green dye can bind DNA and become detectable by fluorescence only when the cell membrane of a cell or within an organism is corrupted. Because of this property, the SYTOX green dye can specifically mark the dead animals in a population.

Here, we showed that the SYTOX green dye can be used as an alternative means of tardigrade viability quantification. Moreover, we monitored the toxicity induced by the SYTOX green and showed that this dye can be used up to 6 days for viability assessment without impairing tardigrade survival.

Effective SYTOX green dye concentrations that have been previously reported vary between 1 and 5 μM for bacteria [29,30] and are up to 1 μM for phytoplankton [31], budding yeast [32] and *Caenorhabditis elegans* as well [34].

In this report, we assessed three different SYTOX green dye concentrations and found that 1 μM can be used without affecting the sensitivity of detection of dead animals in a tardigrade culture. This information is encouraging in consideration of experimental costs. We conclude that the direct observation of tardigrade survival may suffer from underestimation bias and may be greatly improved by the use of the SYTOX green fluorescent dye.

We hope that this new technique for tardigrade viability monitoring may help in the design of future experiments.

## Materials and methods

### Tardigrade handling and culture

*H*. *exemplaris* tardigrades [35] were fed with the unicellular algae *Chlorococcum sp*.; both were purchased from Sciento Company (Manchester, UK) and maintained in culture at 15°C on Chalkley’s medium as previously described [20].

### Sodium azide assay

We used sodium azide (NaN_3_), which is believed to inhibit the activity of mitochondrial cytochrome c oxidase (mitochondria complex IV) by binding metals containing oxygen at the reduction site of the enzyme [36,37]. Sodium azide also inhibits the ATP hydrolase activity of the F-ATPases but not their synthetic activity [38,39]. Groups of 20 adult tardigrade were randomly selected from cultures and disposed in Chalkley’s medium for the control group or in NaN_3_ diluted in Chalkley’s medium to a final concentration of either 5 or 10 mM. Tardigrade without apparent motility for at least 3 minutes after a stimulation by gentle plate soaking were scored as dead by a direct observation under brightfield illumination with a stereomicroscope. Dead animals were scored at either 1 hour, 24 hours, 48 hours or 6 days after sodium azide addition, and plates were stored at 15°C during the experiment duration. Three independent experiments with groups of 20 animals were conducted for each condition. The animals' death scoring was immediately followed by another check by another laboratory experimenter to verify the detection of dead tardigrades.

### SYTOX green assay

We used SYTOX green nucleic acid stain purchased from Molecular Probes (Oregon, USA) [40]. The SYTOX green dye is not fluorescent in aqueous solution and cannot cross either intact cell membranes or egg/embryo shells. When the cell membrane integrity is altered by cell or animal death, SYTOX green can then bind dsDNA, thereby increasing its fluorescence by a great order of magnitude (X1000) and allowing the detection of dead tardigrades by direct fluorescence at a wavelength of 523 nm. Groups of 20 tardigrades were transferred to 12-well plates. The wells were filled with 1.5 ml Chalkley’s medium. SYTOX green was added to each well on the Chalkley’s medium at a final concentration of either 0, 0.1, 1 or 10 μM, and the plates were then incubated at 15°C. Scoring of the dead tardigrades was conducted under a fluorescence microscope at either 1 h, 24 h, 48 h or 6 days as previously described for sodium azide treatments (see previous section). Each experiment was repeated three times. The animals' death scoring was immediately followed by another check by another laboratory experimenter to verify the detection of dead tardigrades.

### Sodium azide assay using SYTOX green fluorescent dye

Adults animals were selected randomly from tardigrade cultures. Groups of 20 animals were incubated in Chalkley’s buffer as control media and in sodium azide-treated media by diluting sodium azide in Chalkley’s buffer to a final concentration of 5 and 10 mM. Tardigrade were deposited on 12-well plates, and wells were supplemented with SYTOX Green at a final concentration of 0, 0.1, 1 or 10 μM in a final volume of 1.5 ml. Counting of living or dead animals was performed after two hours of incubation by a direct observation of their mobility, and photographs were taken at the same time. This operation was repeated 24 hours, 48 hours and one week later. Each sodium azide concentration assay was repeated at least three times.

### Fluorescent microscopy

Microscopic observations were performed using a Leica stereomicroscope M205FCA with a TL3000 Ergo transmitted light base and a Leica DFC3000G camera. Observations and images were made with brightfield illumination, while we used a Leica GFP3 filter (Excitation: 470/440 nm, Emission: 525/550 nm) for the SYTOX green dye fluorescence observations and images. To count the living tardigrades, direct observations were made by verification of motile and non-motile animals under brightfield illumination. The detection of tardigrades associated with a death-linked fluorescence was performed under epifluorescence illumination. Images of both brightfield and SYTOX green fluorescence were taken with a Leica DFC3000G camera.

### Statistical analysis

The pairwise comparisons of the tardigrade death frequency were made using the Z-test with XLSTAT software (Addinsoft, New York, NY, USA).
